# Low Serum Levels of Prealbumin, Retinol Binding Protein, and Retinol Are Frequent in Adult Type 1 Diabetic Patients

**DOI:** 10.1155/2016/2532108

**Published:** 2016-11-29

**Authors:** Luis Forga, Federico Bolado, María José Goñi, Ibai Tamayo, Berta Ibáñez, Carlos Prieto

**Affiliations:** ^1^Department of Endocrinology and Nutrition, Complejo Hospitalario de Navarra, Instituto de Investigación Sanitaria de Navarra (IdiSNA), Calle Irunlarrea 3, Pamplona, 31008 Navarra, Spain; ^2^Department of Digestive System, Complejo Hospitalario de Navarra, Calle Irunlarrea 3, Pamplona, 31008 Navarra, Spain; ^3^Navarrabiomed, Fundación Miguel Servet, Calle Irunlarrea 3, Pamplona, 31008 Navarra, Spain; ^4^Navarrabiomed, Fundación Miguel Servet, Red de Investigación en Servicios Sanitarios en Enfermedades Crónicas (REDISSEC), Calle Irunlarrea 3, Pamplona, 31008 Navarra, Spain

## Abstract

*Aim.* To determine the serum prealbumin (PA), retinol binding protein (RBP), and retinol levels in adult patients with type 1 diabetes (T1D) and to analyze some factors related to those levels.* Methods.* A total of 93 patients (47 women) were studied. Age, gender, BMI, duration of diabetes, chronic complications, HbA1c, lipid profile, creatinine, albumin, PA, RBP, and retinol were recorded. High and low parameter groups were compared by Mann–Whitney *U* and *χ* ^2^ tests. Correlation between parameters was analyzed by Spearman's test. Odds of low levels were analyzed by univariate logistic regression and included in the multivariate analysis when significant.* Results.* 49.5%, 48.4%, and 30.1% of patients displayed serum PA, RBP, and retinol levels below normal values, respectively. A high correlation (Rho > 0.8) between PA, RBP, and retinol serum levels was found. Patients presenting low levels of any of them were predominantly women, normal-weighted, and with lower levels of triglycerides and serum creatinine. No differences in age, macrovascular complications, duration of diabetes, or HbA1c values were observed when comparing low and normal parameter groups.* Conclusion.* Low serum levels of PA, RBP, and retinol are frequent in T1D adult patients. This alteration is influenced by female sex and serum creatinine and triglyceride levels.

## 1. Introduction

Prealbumin (PA) and retinol binding protein (RBP) are proteins primarily synthesized in liver, and their serum levels are used as nutritional status marker of patients. PA and RBP are also believed to be related to the ethiopathogenicity of type 1 diabetes (T1D) [1, 2] and to the derived macrovascular complications [3], respectively. Firstly, PA is a functional constituent of the stimulus-secretion coupling in the healthy beta cell, preserving its functional integrity [1] and protecting it through its action over the immune system [2]. On the other hand, RBP seems to play a protective role in the development of cardiovascular complications, a risk five times higher in the diabetic population, improving the transport of retinol (vitamin A) to places under oxidative stress in order to exert its antioxidant effect [3].

Back in 1985, it was described by the first time that children with T1D displayed lower levels of PA in serum compared to healthy controls [4]. This data was later confirmed in most studies in both children [5–8] and adult [1, 2] T1D populations.

Data about RBP and retinol has proven not to be that consistent: (i) In children with T1D, studies have shown conflicting results. Compared to controls, Baena et al. [3] observed higher RBP levels and lower retinol levels and Espe et al. [9] referred normal RBP and lower retinol levels, while Hozumi et al. [10] reported that no differences could be found among groups. (ii) In adults, it has been described that T1D patients do display lower levels of RBP and retinol than their healthy counterparts [11–13].

Several factors have been hypothesized to be behind the protein levels variations, namely, glycemic control [6, 8, 14], lipid profile [3], changes in main PA circulating form [1] that cause its renal loss [13], and insulin treatment [2, 5]. In this respect, while results are far from being uniform, it is accepted that local low insulin levels in liver do decrease hepatic synthesis of both PA and RBP [2, 3, 6–9, 14].

Apart from those proteins, retinol levels in T1D patients seem to be low [8, 12, 13]. The cause of this fact has been attributed to low carotene to retinol conversion rates in those patients [12, 13] as well as an increased retinol consumption, derived from the increased need of T1D patients to eliminate free reactive oxygen species [8]. However, unlike the case with PA or RBP, no association has been found between glycemic control and retinol levels [8, 9, 12, 13]; instead, a positive correlation was found between retinol and the triglyceride levels [9].

The aim of this study is to analyze if PA and RBP levels, usually employed to evaluate the nutritional state of the people, are modified in adult T1D population by other factors such as glycemic control, sex, BMI, or duration of the disease. Furthermore, we examine the relationship between serum PA, RBP, and retinol levels with diabetic macroangiopathy.

## 2. Materials and Methods

### 2.1. Patients

A total of 93 adult subjects with T1D (46 men and 47 women) who, since 1990, regularly attend the diabetes outpatient clinic of the Complejo Hospitalario de Navarra, in Pamplona, Spain, were studied. In all cases, patients with associated diseases that could alter the results of the analyzed parameters (infections, liver disease, uncontrolled hypothyroidism, nephrotic syndrome, gastrointestinal surgery, and protein-losing enteropathies) were excluded. Twenty-nine patients (31.2%) had been previously diagnosed with primary autoimmune hypothyroidism, eleven (11.8%) with pernicious anemia, thirty (33.3%) with hyperlipemia, and twenty (21.5%) with arterial hypertension. Twelve patients (12.9%) had macroangiopathic complications: 6 with peripheral arterial disease, 5 with ischemic cardiopathy, and 1 with stroke.

Additionally, all patients were under optimized treatment with fast and slow insulin combinations, following an adequate nutrition program and physical exercise supervised by educational nurses and medical specialists. Their dietary intake of proteins was satisfactory. Apart from their corresponding insulin treatment, patients with hypothyroidism or pernicious anemia were also treated with Levothyroxine and vitamin B12, respectively. Additionally, eighteen (19.4%) patients were treated with antihypertensives, 23 (24.7%) with lipid-lowering agents, 10 (10.8%) with platelet aggregation inhibitors (100 mg/day), and 9 (9.7%) with omeprazole.

The study protocol was approved by the regional Ethical Review Board of Navarra, and written informed consent was obtained from all subjects studied before the beginning of the study.

### 2.2. Measurement of Parameters

T1D was diagnosed according to clinical criteria as recommended by the World Health Organization [15]. Parameters such as age, gender, weight, height, BMI, duration of diabetes, chronic complications of diabetes, HbA1c, lipid profile, creatinine, albumin, PA, PTR, and retinol were recorded at the study's day. BMI was calculated using the following formula: weight (in kilograms) divided by height (in meters) squared.

### 2.3. Blood Extraction and Biochemical Parameters

Fasting venous blood was collected and serum was stored at −20°C until further analysis. HbA1c was determined with high-performance liquid chromatography (HPLC; Adams A1c HA, Menarini Diagnostics, Florence, Italy; reference range: 4.1–6.2%). In 2005, the Complejo Hospitalario obtained level II laboratory certification of traceability from the Diabetes Control and Complications Trial (DCCT) reference method through the National Glycohemoglobin Standardization Program.

All biochemical tests were analyzed by the Architect C16000 analyzer (Abbott Diagnostics, Chicago, IL). Total cholesterol and triglycerides (TG) were measured by CHOD-PAP and GPO-PAP, respectively. Serum albumin and creatinine levels were measured using original commercial kits via bromocresol green and photometry methods, respectively.

Serum retinol was determined by HPLC (Reference, Barcelona) normal values (NV): 0.3–1.00 mg/dL; variation coefficient (VC): intra-assay: 5%, interassay: 8.4%. PA and RBP were determined by immunonephelometry (Siemens, BN II Prospec): PA: NV: 20–40 mg/dL; VC: intra-assay 2.7%, interassay 1.1%; RBP: NV: 3–6 mg/dL VC: intra-assay: 3.2%, interassay: 3.2%.

### 2.4. Statistical Analysis

Continuous and categorical variables were compared between normal and low protein groups using Mann–Whitney *U* and *χ* ^2^ tests, respectively. Correlation between quantitative variables was determined using Spearman correlation coefficient.

To assess which characteristics were related with higher risk of having low PA, low RBP, and low retinol levels, univariate logistic regression models were fitted. Additionally, multivariate linear regression was used to simultaneously model all three logarithmically transformed PTR, PA, and retinol levels. All factors that resulted in being significant in the univariate analysis were included as covariates in the model and were maintained when significant. IBM SPSS Statistic 20.0 (SPSS Inc., Chicago, IL) was used for the analyses.

## 3. Results

Main descriptive data from the studied T1D patients are shown in Table 1. Forty-six (49.4%), forty-five (48.4%), and twenty-eight (30.1%) patients displayed serum levels of PA, RBP, and retinol below normal, respectively. Patients presenting low levels of any of the three main parameters under study were predominantly women (63.2% from 51 patients presenting low levels) and normal-weighted (BMI < 25 kg/m^2^) and displayed concomitant low levels of the other two parameters together with creatinine.

Additionally, patients presenting low PA levels had significantly reduced levels of albumin, although serum albumin levels were within normal limits in all cases. Patients with low retinol or RBP levels also displayed lower levels of triglycerides. However, no differences in age, duration of diabetes, glycemic control (HbA1c), or total cholesterol were observed when comparing low and normal parameter groups.

**Figure 1:**
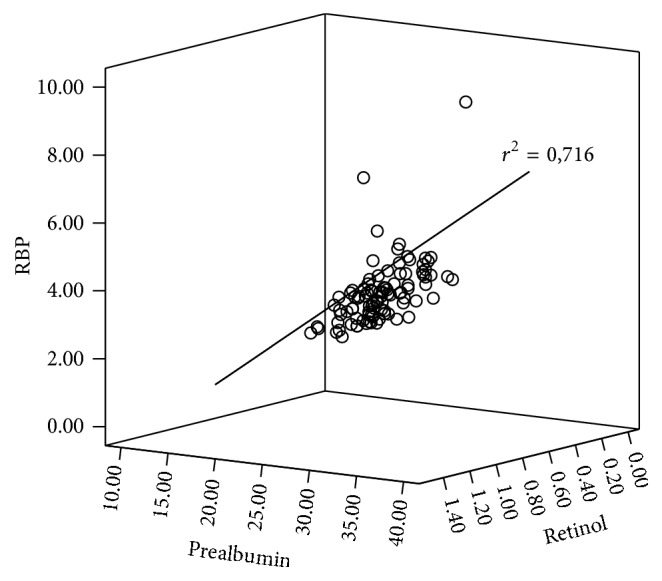
Correlation between PA, RBP, and retinol levels.

BMI correlated weakly with PA (Rho: 0.266; *p*: 0.01), RBP (0.283; *p*: 0.006), retinol (0.251; *p*: 0.015), and creatinine (0.297; *p*: 0.004). A positive correlation was also found between triglycerides and PA levels (0.239; *p*: 0.021), RBP levels (0.358; *p* < 0.001), and retinol levels (0.370; *p* < 0.001). A very strong correlation was found between PA, RBP, and retinol, ranging from Rho values between 0.812 and 0.864; *p* < 0.001 (Figure 1). Additionally, creatinine and PA, RBP, and retinol were highly correlated (Rho from 0.411 to 0.421; *p* < 0.001) (Table  S1, in Supplementary Material available online at http://dx.doi.org/10.1155/2016/2532108).

The univariate analysis (Table 2) revealed that the risk of having low levels of PA was significantly higher for women, patients with normal BMI, and patients with lower levels of creatinine. The risk of having low levels of RBP was increased for women, patients with normal BMI, and patients with lower levels of triglycerides and creatinine. Finally, the parameters significantly associated with the major risk of having low retinol levels were the female sex and the lower triglycerides and creatinine levels.

The multivariate analysis performed to jointly study the effect of covariates on the highly correlated PA, RBP, and retinol revealed that sex and levels of triglycerides and creatinine were independently associated with PA, RBP, and retinol. In this way, female sex and lower triglycerides and lower creatinine plasmatic levels were associated with low PA, RBP, and retinol levels (Supplemental Materials S2 and S3).

No association between TSH and PA, BRP, or retinol levels was found. No association was found also between the concomitant diseases (or their treatments) and the levels of the studied molecules.

No significant differences were found when comparing PA, RBP, and retinol levels between patients with or without diabetic macrovascular complications (Supplemental Material S4).

## 4. Discussion

Approximately half of the T1D adults in our study displayed serum levels of PA or RBP below normal, and virtually one-third of the same population showed low levels of retinol. A high correlation between PA, RBP, and retinol serum levels has been found. Furthermore, those levels seem to be modulated by different factors such as sex, triglycerides levels, and indirect parameters of muscular mass, but association has not been found between RBP or retinol and the presence of diabetic macroangiopathy.

### 4.1. Serum PA, RBP, and Retinol Levels

Except in the paper of Este et al. [9], all published studies coincide in reporting PA levels in the lower normal range in both children and adults, with T1D [1, 2, 5–8]. Our findings match up with published data except in the fact that we do find levels below normality in half of the analyzed patients. This difference might be due to the fact that most of the published studies analyzed children populations of T1D, while our work is focused on adults.

Decreased levels of PA in comparative studies between healthy people and patients with T1D have been attributed to glycemic poor control, meaning that acute [14] or chronic [6, 8] hyperglycemia could negatively affect protein synthesis. However, linear regression analyses do not show any association between HbA1c and PA [1, 6, 8]. In any case, it is possible that insulin, as an important protein metabolism regulator, could cause the observed alteration at a level of protein synthesis [5, 6, 8], due to a lower local hepatic concentration, nonphysiological continuous administrations in T1D patients, or fluctuations in plasmatic concentration.

In healthy people, PA has a complex equilibrium between different quaternary structures in serum but exists mainly as a tetramer of 14-kDa subunits with only a small amount of PA monomer present in vivo. So, measurements of PA in serum by conventional methods mainly reflect the tetrameric form. Another hypothesis about the lowered PA levels proposes that the concentration of tetrameric form of PA is decreased in T1D, whereas that of the monomeric form is increased [1].

Studies about RBP levels have revealed contradictory results in children. When comparing T1D children with control groups, Baena et al. [3] describe higher levels, Espe et al. [9] and Gebre-Medhin et al. [16] describe similar levels, and Kemp et al. [8] describe lower levels. Focused on T1D adults, the only studies published agree with our work in showing decreased RBP levels [11, 17]. No relation has been described between metabolic control or duration of diabetes and RBP levels [8, 9], resulting in the same attribution as that in the case of PA levels: a deficient protein synthesis caused by a hepatic hypoinsulinism [8].

Regarding the metabolism of retinol, it has been suggested that the hepatic mobilization of vitamin A and its intestinal conversion from carotene to retinol are decreased, resulting in lower levels of both retinol and RBP in T1D patients [12, 13].

In order to explain the relationship between the three molecules, we have to consider that retinol is stored in the form of retinol esters in the liver, and, before release, these esters are hydrolyzed, and the free alcohol is linked to a specific protein, RBP. The retinol-RBP compound is then secreted into the blood flow, where it is bound to PA by means of a noncovalent link [3]. Therefore, the observed correlations between RBP and retinol levels [3, 9], between PA and RBP levels [8], and also between the three molecules PA, RBP, and retinol are not surprising [17]. Our results agree with the latter, as we have found a significant and strong correlation over 0.8.

### 4.2. Factors Related with Risk of Having Low PA, RBP, and Retinol Levels

Kobbah et al. [7] described that levels of PA were normalized during follow-up in boys with T1D, while girls maintained low PA levels. This is the only work pointing sex related differences in this protein levels. Espe et al. [9] in children and Basu et al. [12] in young adults did not observe those differences. In our study, the risk of presenting low PA levels in women is increased fourfold. Additionally, being woman does also increase the risk of having low RBP levels 3.6-fold and the risk of having low levels of retinol 4.5-fold.

On the other hand, a positive correlation between RBP or retinol levels and triglycerides has been described [3, 9]. This led to another hypothesis where retinol levels in T1D patients would be lowered because of a protective role of RBP in case of dyslipidemia. In this hypothesis, RBP would act transporting the antioxidant retinol to places under oxidative stress in order to eliminate reactive oxygen species and protect from atherogenesis [3]. Our data does not support this hypothesis, since no association has been found between RBP or retinol and the presence of diabetic macroangiopathy.

In our study, BMI was also found to be in relation with having low levels of PA or RBP proteins. In the absence of overweight or obesity, the risk of having low PA levels is increased 2.3-fold; this value is increased to 2.59 if we analyze the risk of having low RBP levels. BMI is a fat mass indicator, but it also reflects muscle mass [18], that is lower in women than in men [19]. Furthermore, serum levels of PA [20] and creatinine [21] are correlated to muscle mass. These data, all together, suggests that the lower the BMI, the lower the muscle mass and, therefore, the lower the levels of PA and creatinine. Our findings of the univariate analysis support this theory, because the risk of having low PA decreases by 31% for each creatinine increment level of 0.1 mg/dL. For the same creatinine increment, the risk of low RBP decreases by 36% while in the case of retinol it drops below 50%. Noteworthily, the creatinine, together with triglycerides and sex, stands up in the multivariate analysis as an independent factor that affects all three PA, RBP, and retinol.

None of the patients showed albumin levels below normal values. However, in agreement with several works [4, 5, 7, 14, 22], the ones with low PA had lower albumin levels.

In regard to thyroid function, hypothyroidism has been described to be associated with decreased retinol levels independently of PA and RBP levels [23]. Additionally, with PA being one of the thyroxine transporting proteins [1], we analyzed the relation between TSH and PA and RBP and retinol levels, finding no correlation between those levels.

One of the strengths of our study resides in the number of T1D patients included in comparison with previous works, more so being adult T1D patients. This fact allowed us to achieve original results unravelling the interaction between factors. However, there have been some limitations since muscle mass has been indirectly estimated from BMI and creatinine levels.

To summarize, nearly one half of T1D patients under nutritional supervision, that follow a proper diet for their pathology, display low PA and RBP levels, which suggests that these levels might not reflect the actual nutritional status of the T1D patients. There is a high correlation between PA, RBP, and retinol levels, which are strongly influenced by sex, muscular mass, and serum triglyceride levels. While we have not found any relation between retinol levels and macroangiopathy, more studies are needed in order to analyze the consequences that the decreased levels of these molecules might have in patients with T1D and their causes.

## Supplementary Material

Supplementary material shows the relation between relevant variables and macrovascular complications and their impact over the multivariate model. Briefly, Table S1 shows Spearman's correlation between variables. Table S2 shows the significant variables in the multivariate analysis. Table S3 shows the independent impact of each variable on the multivariate model and Table S4 shows the relationship between diabetic macroangiopathy and the three studied molecules.

## Figures and Tables

**Table 1:** Clinical characteristics of all patients, for patients stratified by low or normal PA, RBP, and retinol levels and group comparisons.

Parameters	All patients	PA			RBP			Retinol		
		Low	Normal	*p* value	Low	Normal	*p* value	Low	Normal	*p* value
Age (years)	45.3 (11.6)	46.2 (11.9)	44.5 (11.4)	0.556	45.0 (12.1)	45.7 (11.2)	0.738	45.4 (11.8)	45.3 (11.6)	0.957
Duration of DM (years)	20.6 (11.2)	21.3 (11.4)	20.0 (11.0)	0.489	19.8 (10.8)	21.4 (11.6)	0.491	19.9 (9.7)	20.9 (11.8)	0.801
HbA1c (%)	7.8 (0.9)	8.0 (1.1)	7.7 (0.6)	0.195	7.8 (1.0)	7.7 (1.0)	0.929	7.9 (1.0)	7.7 (1.0)	0.306
Total cholesterol (mg/dL)	179.6 (33.8)	178.3 (30.4)	180.8 (37.1)	0.642	177.1 (34.1)	181.9 (33.7)	0.620	179.2 (28.4)	179.7 (36.1)	0.769
Triglycerides (mg/dL)	66.9 (32.1)	61.3 (20.8)	72.2 (39.9)	0.402	59.9 (26.5)	73.5 (35.7)	0.031	53.0 (15.6)	72.9 (35.5)	0.003
Albumin (g/dL)	4.4 (0.1)	4.3 (0.3)	4.5 (0.2)	0.002	4.3 (0.3)	4.4 (0.3)	0.543	4.3 (0.3)	4.4 (0.3)	0.248
PA (mg/dL)	20.7 (4.7)	17.1 (2.2)	24.2 (3.7)	<0.001	17.4 (7.1)	23.8 (4.1)	<0.001	16.7 (2.6)	22.4 (4.3)	<0.001
RBP (mg/dL)	3.2 (1.5)	2.5 (0.5)	3.8 (1.3)	<0.001	2.3 (0.4)	3.9 (1.2)	<0.001	2.2 (0.4)	3.6 (1.2)	<0.001
Retinol (mg/dL)	0.4 (0.2)	0.3 (0.1)	0.5 (0.2)	<0.001	0.3 (0.1)	0.5 (0.2)	<0.001	0.2 (0.1)	0.4 (0.1)	<0.001
Creatinine (mg/dL)	0.8 (0.1)	0.8 (0.1)	0.9 (0.3)	0.011	0.8 (0.1)	0.9 (0.3)	0.003	0.7 (0.1)	0.9 (0.3)	<0.001
Sex				0.002			0.004			0.002
Female	50.5%	66.0%	34.0%		63.8%	36.2%		44.7%	53.3%	
Male	49.5%	32.6%	67.4%		32.6%	67.4%		15.2%	84.4%	
BMI (kg/m^2^)				0.047			0.026			0.098
BMI ≥ 25	45.2%	61.9%	38.1%		35.7%	64.3%		21.4%	78.6%	
BMI < 25	54.8%	41.2%	58.8%		58.8%	41.2%		37.3%	62.7%	

Data summarized using mean (standard deviation) except for sex and qualitative BMI, for which percentage by row is given. PA: prealbumin, RBP: retinol binding protein.

**Table 2:** Univariate logistic regression results to assess association with low PA, RBP, or retinol levels.

	PA		RBP		Retinol	
	OR (95% CI)	*p* value	OR (95% CI)	*p* value	OR (95% CI)	*p* value
Sex						
Male	Reference		Reference		Reference	
Female	4.00 (1.69–9.49)	0.001	3.65 (1.55–8.59)	0.002	4.5 (1.67–12.10)	0.002
BMI						
BMI ≥ 25	Reference		Reference		Reference	
BMI <25	2.32 (1.01–5.36)	0.046	2.57 (1.11–5.97)	0.026	2.18 (0.86–5.52)	0.095
Triglycerides (10 mg/dL)	0.89 (0.78–1.03)	0.103	0.86 (0.74–0.99)	0.035	0.73 (0.57–0.92)	0.001
Creatinine (0.1 mg/dL)	0.69 (0.51–0.94)	0.005	0.64 (0.46–0.89)	0.001	0.48 (0.31–0.73)	<0.001

PA: prealbumin, RBP: retinol binding protein.
