# Supplementary material for: Low Serum Levels of Prealbumin, Retinol Binding Protein, and Retinol Are Frequent in Adult Type 1 Diabetic Patients
Source: J Diabetes Res. 2016 Nov 29;2016:2532108. doi: 10.1155/2016/2532108 (PMC5153501; doi:10.1155/2016/2532108)
Supplement: Supplementary file 1 — Supplementary material shows the relation between relevant variables and macrovascular complications and their impact over the multivariate model. Briefly, Table S1 shows Spearman's correlation between variables. Table S2 shows the significant variables in the multivariate analysis. Table S3 shows the independent impact of each variable on the multivariate model and Table S4 shows the relationship between diabetic macroangiopathy and the three studied molecules. [file 2532108.f1.doc]

Table S1: Spearmans correlation between variables

| Spearman's Rho | BMI | HbA1c | Cholesterol | Triglycerides | Albumin | Prealbumin | RBP | Retinol |
| --- | --- | --- | --- | --- | --- | --- | --- | --- |
| BMI |  |  |  |  |  |  |  |  |
| HbA1c | -0.073 |  |  |  |  |  |  |  |
| Cholesterol | 0.011 | 0.049 |  |  |  |  |  |  |
| Triglycerides | 0.181 | 0.123 | 0.398^**^ |  |  |  |  |  |
| Albumin | -0.026 | -0.144 | 0.081 | -0.057 |  |  |  |  |
| Prealbumin | 0.266^**^ | -0.162 | 0.1 | 0.239^*^ | 0.272^**^ |  |  |  |
| RBP | 0.283^**^ | -0.005 | 0.082 | 0.358^**^ | 0.076 | 0.864^**^ |  |  |
| Retinol | 0.251^*^ | -0.13 | 0.108 | 0.370^**^ | 0.16 | 0.812^**^ | 0.833^**^ |  |
| Creatinine | 0.297^**^ | -0.273^**^ | -0.134 | 0.027 | 0.099 | 0.411^**^ | 0.414^**^ | 0.421^**^ |
| **p<0.01 |  |  |  |  |  |  |  |  |
| *p<0.05 |  |  |  |  |  |  |  |  |

Table S2: Multivariate results assessing association with log-transformed PA, RBP and retinol levels

| **Effect** | **Wilks' Lambda value** | **F** | **p Value** |
| --- | --- | --- | --- |
| Intercept | 0.015 | 1844.440 | <0.001 |
| Triglycerides | 0.748 | 9.765 | <0.001 |
| Creatinine | 0.683 | 13.445 | <0.001 |
| Sex | 0.855 | 4.924 | 0.003 |

Table S3: Analysis of variance of the multivariate model

|  | **Dependent Variable (log-transformed)** | **F** | **p Value** |
| --- | --- | --- | --- |
| Corrected model | PTR | 28.118 | <0.001 |
|  | PA | 17.204 | <0.001 |
|  | Retinol | 23.013 | <0.001 |
| Intersection | PTR | 14.539 | <0.001 |
|  | PA | 1332.154 | <0.001 |
|  | Retinol | 267.386 | <0.001 |
| Triglycerides | PTR | 22.928 | <0.001 |
|  | PA | 9.608 | 0.003 |
|  | Retinol | 22.433 | <0.001 |
| Creatinine | PTR | 34.97 | <0.001 |
|  | PA | 13.54 | <0.001 |
|  | Retinol | 25.105 | <0.001 |
| Sex | PTR | 7.426 | 0.008 |
|  | PA | 14.647 | <0.001 |
|  | Retinol | 6.67 | 0.011 |
